# Increase in child behavior problems among urban Brazilian 4-year olds: 1993 and 2004 Pelotas birth cohorts

**DOI:** 10.1111/jcpp.12236

**Published:** 2014-04-16

**Authors:** Alicia Matijasevich, Elizabeth Murray, Alan Stein, Luciana Anselmi, Ana M Menezes, Iná S Santos, Aluísio JD Barros, Denise P Gigante, Fernando C Barros, Cesar G Victora

**Affiliations:** 1 Postgraduate Program in Epidemiology, Federal University of PelotasPelotas, Brazil; 2 Department of Preventive Medicine, School of Medicine, University of São PauloSão Paulo, Brazil; 3 Section of Child and Adolescent Psychiatry, Department of Psychiatry, Oxford UniversityOxford, UK; 4 Postgraduate Program in Health and Behavior, Catholic University of PelotasPelotas, Brazil

**Keywords:** Mental health, behavior problems, child behavior check list, longitudinal studies

## Abstract

**Background:**

There are an increasing number of reports on time trends in child and adolescent psychological problems but none from low- and middle-income countries, and very few covering the preschool period. The aim was to investigate changes in preschool behavioral/emotional problems in two birth cohorts from a middle-income country born 11 years apart.

**Methods:**

We analyzed data from the 1993 and 2004 Pelotas birth cohort studies from Brazil. A subsample of 4-year olds from the 1993 cohort (634) and all 4-year olds from the 2004 cohort (3750) were assessed for behavioral/emotional problems through maternal report using the Child Behavior Checklist (CBCL). Response rates in these two population-based cohorts were above 90%.

**Results:**

We found a significant increase in CBCL total problems, internalizing and externalizing mean scores over the 11-year period. For 1993 and 2004 Pelotas cohorts, respectively, CBCL mean values (*SE*) total problems scores were 27.9 (0.8) and 34.7 (0.3); for internalizing scores, 5.7 (0.2) and 6.3 (0.1) and for externalizing scores, 12.4 (0.4) and 15.5 (0.1). After adjusting for confounding variables, the largest increase from 1993 to 2004 was identified in the aggressive behavior syndrome score (Cohen's *d *=* *.50), followed by the externalizing problem score (Cohen's *d *=* *.40) and CBCL total problem score (Cohen's *d *=* *.36), respectively. The rise in child psychological problems was more marked in children from families with fewer assets and with less educated mothers.

**Conclusions:**

Our findings provide evidence for a substantial increase in preschool behavioral problems among children in Brazil over an 11-year period.

## Introduction

Mental illnesses such as depression, alcohol use disorders, and psychoses are among the 20 leading causes of years lost due to disability (Schmidt, Duncan, Azevedo e Silva, Menezes, Monteiro, Barreto, Chor, & Menezes 2012). These conditions often have their origins in childhood and adolescence, run throughout the life cycle, are associated with a wide range of adverse outcomes in adult life, and may even jeopardize the well-being of future generations (Angold & Costello, 1995; Fergusson, Horwood, & Ridder, 2005, 2007; Richards et al., 2009). A systematic review (Kieling et al., 2011) of studies from low- and middle-income countries showed a prevalence of mental health problems in children and adolescents of about 10–20%, with anxiety, conduct, attention, and depressive disorders being the most common.

In the last few decades, time trend analyses have suggested increases in mental health problems among children and adolescents from high-income countries. Achenbach and Howell (1993) found increases in Child Behavior Checklist (CBCL) problem scores in American children aged 7–16 years between 1976 and 1989. Even though those scores decreased slightly between 1989 and 1999, they remained higher than those seen in 1976 (Achenbach, Dumenci, & Rescorla, 2003). Among UK adolescents aged 15–16 years, conduct problem scores increased markedly across all social class categories between 1974 and 1999 (Collishaw, Maughan, Goodman, & Pickles, 2004). Recent comparisons between 1986 and 2006 in the same age group showed a modest increase in parent-reported youth conduct problems, but a substantial increase in adolescent emotional problems (Collishaw, Gardner, Maughan, Scott, & Pickles, 2012; Collishaw, Maughan, Natarajan, & Pickles, 2010).

Researchers from other countries have also found rising rates of behavioral problems, although not as marked as in the studies conducted in North America or the United Kingdom. Tick and colleagues (Tick, van der Ende, & Verhulst, 2007a) studied trends in emotional and behavioral problems in Dutch children aged 6–16 years between 1983 and 2003. They found small increases in the mean population levels of parent-reported problems in children; such changes were most marked between 1993 and 2003.

There has been very little research in the preschool years. One Dutch study of children aged 2–3 years, however, showed a small decline in parental reports of emotional and behavioral problems between 1989 and 2003 (Tick, van der Ende, Koot, & Verhulst, 2007b).

Ninety per cent of the world's children and adolescents live in low- and middle-income countries. However, few epidemiological studies have been conducted to assess time trends in the mental health of children and adolescents in these countries (Kieling et al., 2011). It was possible to find only one previous study that examined time trends in the prevalence of child mental health problems from a low-income country. The study was carried out in an urbanized part of Khartoum, Sudan, among children in the age range 3–15 years (Rahim & Cederblad, 1984). Even though authors reported that boys of school age in 1980 tripled their rate of behavioral symptoms, this increase was not seen among girls and boys under 7. As prevalence rates of child and adolescent psychopathology tend to vary markedly between countries (Kieling et al., 2011; Roberts, Attkisson, & Rosenblatt, 1998), more studies are needed to examine trends in low- and middle-income countries to see if the pattern of change is comparable to that identified in high-income countries.

The aim of this study was to examine changes in behavioral and emotional problems among children aged 4 years, over an 11-year period, using data from the 1993 and 2004 Pelotas Brazilian birth cohort studies. Furthermore, we aimed to examine whether changes in emotional and behavioral problems could be accounted for by differences in maternal and child characteristics between cohorts.

## Methods

### Research setting and study design

The city of Pelotas is located in Southern Brazil. It is a medium-sized city with a population of about 340,000 inhabitants according to the 2000 Brazilian Population Census (Brazilian Demographic Census, IBGE, 2000). Its population is predominantly urban (93.3%) and more than 99% of all birth deliveries take place in hospitals.

During the whole years of 1993 and 2004, all mothers giving birth to children who resided in the urban area of the city of Pelotas were approached to have their children included in a birth cohort study. Data were collected directly by the study teams using consistent methodology (5304 and 4287 births in 1993 and 2004 cohorts, respectively). The nonresponse rate at recruitment in both cohorts was below 1%. Soon after delivery, mothers were interviewed using a structured questionnaire. Detailed information was collected on demographic, socioeconomic, behavioral and biological characteristics, reproductive history, and health care utilization. The cohort children were followed up at several time points, when data were collected on growth, morbidity, development, and feeding habits, as well as social demographic and family data.

In the 1993 cohort, all low birthweight children plus a random sample of 20% of the rest of the sample were visited at home at approximately 48 months postnatally (*M *=* *54.1, *SD *= 3.7). Of the intended sample (1460 children), 87.2% were located. Half of this subsample (*n *=* *636) were randomly selected for the purposes of this study, to investigate behavior and emotional problems in 4-year-old children, of whom 634 were located.

In the 2004 cohort study, all cohort children were sought when they were approximately 48 months old (*M *=* *49.5, *SD *= 1.7) and 3799 children were located. Only 49 families declined to participate in the study (response rate of 91%). A detailed description of the methodology of the 1993 and 2004 Pelotas cohort studies is given elsewhere (Santos et al., 2011; Victora et al., 2008).

### Assessment of behavior problems at 4 years of age

Child behavioral/emotional problems were assessed in the 1993 and 2004 cohorts using the Child Behavior Checklist (CBCL) (Achenbach, 1991). We used the 4–18 year version, the only version validated in Brazil, in both cohorts to ensure comparability of the two studies. The same psychologist (LA) trained the interviewers and supervised the application of the test in both cohorts. The 118 behavioral and emotional items of the CBCL were scored by mothers. If the mother was unavailable, the assessment was not conducted. A profile of childhood psychological problems provided scores on eight empirically derived scales: withdrawn, somatic complaints, anxious/depressed, social problems, thought problems, attention problems, aggressive behavior, and rule-breaking behavior. Data from these scales were summed to provide an overall score (total problems), and were also grouped in two broad dimensions (internalizing and externalizing problems). Several studies have supported the CBCL's psychometric properties, showing good reliability and validity in both clinical and nonclinical populations. The CBCL was validated among Brazilian children by Bordin, Mari, and Caeiro (1995)

The CBCL is designed to be self-completed (by parents); however, to maximize response rates, standardization, accuracy, and to prevent embarrassment or error among less literate mothers than others, questions were read out by a trained interviewer in the identical order and form as in the original CBCL. The administration of CBCL as an interview is accepted by the instrument's authors Achenbach (1991).

### Demographic information

Information on socioeconomic status (SES), marital status, maternal age, maternal skin color, parity, maternal smoking during pregnancy, and child characteristics was collected in the perinatal interview. Maternal employment was assessed at the 4-year follow-up.

To measure socioeconomic position, asset indices were constructed for each cohort, based on principal components analysis of the ownership of domestic goods (e.g., household appliances, car) and characteristics of the residence (e.g., number of bathrooms, presence of a maid). For analyses, the first principal component was used and expressed in quintiles.

Information on maternal formal education was collected as number of completed years of formal education, and categorized as: 0–4, 5–8, and ≥9 years.

Women who were single, widowed, divorced, or lived without a partner were classified as single mothers. Mother's skin color was self-reported and categorized as white or black/mixed. Maternal age was collected in complete years and categorized as ≤19, 20–34, and ≥35 years. Parity was defined as the number of previous viable pregnancies and categorized as 0, 1, and ≥2. Maternal smoking behavior during pregnancy was assessed retrospectively at birth and was based on mother's report. Women who smoked at least one cigarette per day, everyday in any trimester of pregnancy, were categorized as regular smokers. Women that smoked less than one cigarette everyday were classified as nonsmokers.

Infant variables were preterm birth (<37 weeks gestation), low birthweight (<2,500 g), sex, and multiple pregnancy. Birth weight was recorded by hospital staff. Gestational age was estimated based on the last menstrual period (LMP) whenever it was consistent with birthweight, length, and head circumference. If the LMP-based gestational age was unknown or inconsistent, the clinical maturity estimate based on the Dubowitz method was used (Dubowitz, Dubowitz, & Goldberg, 1970), which was performed on almost all newborns.

In both cohorts, maternal psychiatric problems were assessed using the Self-Report Questionnaire of Minor Psychiatric Disorders (SRQ). The cutoff point adopted was 8 as suggested by a validation study in a Brazilian sample (Mari & Williams, 1986). The assessment took place when the child was 4 years old in the 1993 cohort, and when the child was 3 months old in the 2004 cohort.

Maternal employment was defined as work outside the home and was categorized as: never worked, always worked, and worked sometime during the first 4 years of the child's life.

### Statistical analyses

Differences in the distribution of exposure variables between cohorts were assessed using Pearson *χ*^2^ tests and *χ*^2^ tests for linear trend. To address the oversampling of low birthweight children in the 1993 cohort, the data were weighted using probability weights of .33 for overrepresented low birthweight children and 1.28 for the rest of the sample (Dorofeev & Grant, 2006). This technique statistically adjusts the proportions of the 1993 sample to match the total 1993 cohort, and has been an accepted method for many publications in this cohort (Barros et al., 2012; Brion et al., 2010, 2011; Matijasevich et al., 2011; Victora et al., 1998). The weighted 1993 estimates were used in all subsequent analyses.

The CBCL scores were analyzed as a continuous outcome in original score units, whereby a higher score reflects greater problems in that domain. The difference in mean age at follow-up (in months) between the 1993 cohort (*M *=* *54.1, *SD*=3.7) and the 2004 cohort (*M *=* *49.5, *SD*=1.7) was controlled for by the inclusion of age as a covariate in all analyses.

To assess the effects of maternal and child characteristics on CBCL scores across both cohorts, and to examine differences in the confounding structures between the 1993 and 2004 cohorts, a multivariable ANOVA was conducted. This included all maternal and child characteristics in a single model, to account for multiple comparisons, and was repeated for each of the CBCL outcomes. Post hoc analyses of simple effects were conducted using univariable ANOVAs, and Bonferroni adjustments were made where appropriate.

ANCOVA analyses were conducted to assess whether differences in CBCL scores between the 1993 and 2004 cohorts could be attributable to broader changes in the demographic characteristics of the samples. For an exposure variable to be included in the model, it had to either have a significant effect on CBCL scores and have a significantly different distribution between the 1993 and 2004 cohorts, or have a differential effect on CBCL scores across cohorts, indicated by the presence of a significant confounder*cohort interaction. On the basis of these criteria, covariates selected for the model included: assets index, maternal schooling, marital status, maternal age, maternal smoking during pregnancy, maternal employment, maternal psychiatric problems, low birthweight, and multiple pregnancy.

Successive ANCOVA analyses were conducted by incorporating additional confounders into each consecutive model to assess the additive effect of each set of factors. ANCOVAs were conducted in the following order: (a) adjusting for age at time of testing; (b) adjusting for family socioeconomic position; (c) adjusting for maternal characteristics; (d) adjusting for child characteristics; (e) adjusting for maternal employment and maternal psychiatric problems. Each successive adjustment included all adjustments in previous models.

To determine the magnitude of change in CBCL scores, effect sizes (Cohen's *d* and effect size *r*) were calculated for the change in CBCL scores from 1993 to 2004.

For confirmatory purposes, the full analysis was repeated using a dichotomous classification of CBCL scores, comparing clinical and nonclinical groups. The clinical group was identified as those children with a *t* score higher than 63 in the CBCL total, externalizing or internalizing scales, and higher than 70 points in the individual syndrome scales, in accordance with the CBCL manual (Achenbach, 1991).

All analyses were performed using Stata version 12.0 software (College Station, TX).

The study protocol was approved by the Medical Ethics Committee of the Federal University of Pelotas, affiliated with the Brazilian Federal Medical Council. In 1993, verbal consent was obtained from mothers, and in 2004, a signed informed consent form was used, after informing mothers of the study objectives.

## Results

### Differences between the included and nonincluded children in the 1993 cohort

As expected due to the sampling method, low birthweight, preterm birth, and multiple births were characteristics more frequent among the children included in the 1993 sample. No differences were found regarding socioeconomic and demographic characteristics (ethnic origin, family income, maternal schooling, marital status, age or smoking during pregnancy, and child's sex) (data not shown, available on request). After weighting, the included sample was matched on all characteristics to the nonincluded cases in the 1993 cohort, indicating that the weighted sample was representative of the cohort as a whole (data not shown, available on request).

### Changes in maternal and child characteristics between the 1993 and 2004 cohorts

Differences in maternal and child characteristics between the 1993 (weighted sample) and 2004 cohorts are presented in Table S1 (available online). The 2004 cohort had a higher frequency of women with more years of schooling, more single mothers, more adolescent mothers (<20 years), more women with a higher prevalence of psychiatric problems, more primiparous women, and a higher number of women who were employed in the first 4 years of the child's life. No difference was observed between the two cohorts regarding maternal skin color or maternal smoking during pregnancy. The proportion of preterm birth was higher in 2004 cohort than in 1993 cohort; however, no difference was observed in low birthweight or multiple pregnancies.

### Changes in behavioral/emotional problems among children between 1993 and 2004

**Figure 1:**
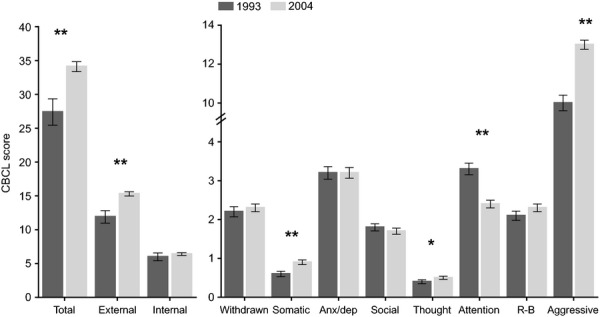
Mean CBCL scores and *SEM* in girls in the 1993 and 2004 Pelotas cohort studies adjusted for age (months) at the time of testing. Note. *p <* *.05, **p <* *.001; R-B, rule-breaking behavior; *SEM,* standard error of the mean

**Figure 2:**
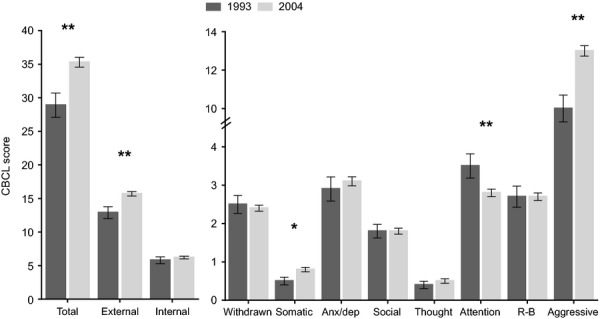
Mean CBCL scores and *SEM* in boys in the 1993 and 2004 Pelotas cohort studies adjusted for age (months) at the time of testing. Note. *p <* *.05, **p <* *.001; R-B, rule-breaking behavior; *SEM,* standard error of the mean

Changes in CBCL scores between the 1993 and 2004 cohorts are presented in Figures1 and 2 (among girls and boys, respectively). Substantial increases were detected in mean CBCL total problems, internalizing and externalizing mean scores (approximately 25%, 10%, and 25%, respectively). Increases were also identified in mean somatic complaints, thought problems (only among girls), and aggressive behavior syndrome scores. Interestingly, there was a reduction in attention problem mean scores over the study period (approximately 21%). There were no significant differences in the withdrawn, anxious/depressed, social problems, and rule-breaking behavior syndrome scores between the two cohorts.

### Effects of maternal and child characteristics on behavioral/emotional problems

Multivariable ANOVAs revealed that a number of maternal and child characteristics affected CBCL scores across both cohorts; however, these varied according to CBCL syndrome (Table1). CBCL total score was identified to be significantly affected by maternal schooling, marital status, maternal age, maternal smoking during pregnancy, and maternal psychiatric problems. The internalizing problem score was additionally affected by parity, and the externalizing problem score was also affected by sex of the child.

**Table 1:** Multivariable ANOVA main effects (*F* statistic) of familial, maternal, and child characteristics on CBCL scores across both cohorts

Variables	CBCL total	Internal	External	Withdrawn	Somatic	Anx./Dep.	Social	Thought	Attention	Rule-breaking	Aggressive
Family assets	0.56	1.07	0.62	1.06	1.30	1.91	0.96	0.77	0.50	2.22	0.46
Maternal schooling	3.01*	4.66*	1.67	2.41	0.03	5.50*	3.92*	2.69	4.87*	1.67	1.31
Marital status	6.33*	6.05*	6.96*	7.81*	0.45	4.67*	2.97	1.38	1.72	1.19	8.68*
Maternal age	4.13*	1.34	6.84*	3.16*	0.44	0.41	4.00*	0.56	2.27	2.65	7.31*
Maternal skin color	2.43	1.40	1.54	0.30	0.96	2.52	1.33	0.75	5.88*	0.67	2.03
Parity	1.54	4.10*	0.07	6.99*	1.70	3.49*	1.81	0.02	3.26*	0.13	0.14
Maternal smoking	32.83**	2.32	58.47**	3.45	0.01	0.13	6.57*	5.22*	33.28**	40.97**	47.93**
Maternal employment	1.05	1.17	0.50	0.05	2.04	2.50	5.22*	3.45*	0.83	0.26	0.58
Maternal psychiatric	195.85**	205.51**	139.33**	115.51**	53.93**	167.74**	78.86**	66.04**	119.13**	58.79**	125.00**
Preterm birth	2.21	1.01	0.56	1.05	0.19	0.66	0.98	0.43	1.94	0.01	0.32
Low birthweight	0.13	0.01	0.51	0.03	0.47	0.32	0.16	0.56	0.33	0.05	0.56
Child's sex	2.82	2.08	5.19*	2.58	1.35	6.86*	1.45	0.26	7.16*	28.47**	0.43
Multiple pregnancy	0.01	0.01	0.29	1.01	1.03	0.07	4.59*	3.91*	0.01	0.73	0.63

Controlling for age at follow-up and using weighted 1993 scores

* *p *<* *.05

** *p *<* *.001.

Post hoc comparisons using univariable ANOVAs (Table S2) identified a higher score in at least one CBCL syndrome in children of mothers who had less schooling, were single mothers, were adolescent at time of birth, had black/mixed skin color, were multiparous (≥2 previous viable pregnancies), smoked during pregnancy, were unemployed, and had psychiatric problems. Girls were identified as having higher scores in the anxious/depressed syndrome, but lower scores in the syndromes of attention problems and rule-breaking behaviors.

**Figure 3:**
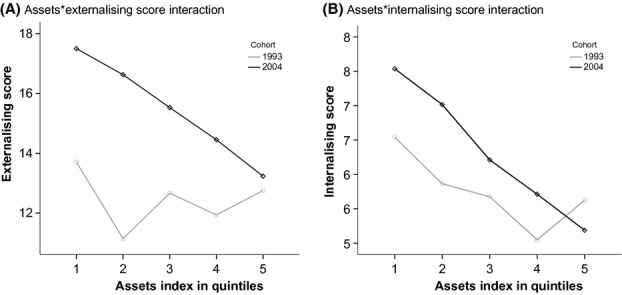
Plot of assets*externalizing score (a) and assets*internalizing score (b) interactions in the 1993 and 2004 Pelotas cohort studies

The presence of significant cohort interactions revealed differences in the effect of some child and maternal characteristics on CBCL scores between cohorts (Table S3). Interaction effects were most consistently identified between cohort and family assets (Figure3), maternal schooling and maternal psychiatric problems (Figures S1a–S1d). CBCL score increases between 1993 and 2004 were more observable in children from families in the lower asset quintiles (compared with the highest asset quintile) and in children with less educated mothers. Children from families in the highest asset quintile and with highly educated mothers showed almost no increase in behavioral problems between 1993 and 2004.

To assess whether broad changes in maternal and child characteristics between 1993 and 2004 could account for the observed change in CBCL scores, successive ANCOVAs were conducted (Table2). Accounting for changes in family SES between 1993 and 2004 (Model 2) did not explain the differences in CBCL scores, with the exception of the anxious/depressed syndrome. Following adjustment for SES, this syndrome was identified to be significantly higher in the 2004 cohort compared with the 1993 cohort.

**Table 2:** ANCOVA analyses of differences in CBCL scores between the 1993 and 2004 cohorts

CBCL Scales	Model 1		Model 2		Model 3		Model 4		Model 5	
	*F*	*p*	*F*	*p*	*F*	*p*	*F*	*p*	*F*	*p*
Total problems	59.73	<.001**	67.06	<.001**	65.74	<.001**	65.68	<.001**	69.05	<.001**
Internalizing	6.32	.012*	10.25	.001*	8.86	.003*	8.97	.003*	8.76	.003*
Externalizing	62.06	<.001**	68.85	<.001**	64.46	<.001**	64.20	<.001**	65.91	<.001**
Withdrawn	0.41	0.524	1.32	0.250	0.83	.364	0.86	0.355	0.18	.667
Somatic	15.18	<.001**	15.94	<.001**	14.89	<.001**	15.19	<.001**	15.64	<.001**
Anxious/Depressed	3.55	0.060	6.73	.010*	5.93	.015*	5.94	.015*	7.10	.008*
Social	0.16	0.692	1.19	.276	0.72	.397	0.74	.389	1.10	.294
Thought	19.03	<.001**	19.59	<.001**	18.18	<.001**	18.46	<.001**	18.15	<.001**
Attention	28.45	<.001**	25.20	<.001**	29.20	<.001**	29.01	<.001**	29.47	<.001**
Rule-breaking	0.11	.744	0.52	.471	0.14	.708	0.13	.714	0.11	.740
Aggressive	102.44	<.001**	108.82	<.001**	104.03	<.001**	103.69	<.001**	106.43	<.001**

* *p *<* *.05

** *p *<* *.001.

Model 1. Adjusted for age at the time of CBCL assessment.

Model 2. Adjusted for model 1 plus family socioeconomic status (assets index and maternal education).

Model 3. Adjusted for model 2 plus maternal characteristics (marital status, maternal age, parity, maternal smoking during pregnancy).

Model 4. Adjusted for model 3 plus child characteristics (low birthweight and multiple pregnancy).

Model 5. Adjusted for model 4 plus maternal employment and maternal psychiatric problems.

Further controlling for variation in maternal characteristics (Model 3), child characteristics (Model 4), and maternal employment and psychiatric problems (Model 5) did not account for the observed changes in CBCL scores between 1993 and 2004, suggesting that the changes in CBCL scores are independent of variation in the sample characteristics.

After adjusting for all confounding variables, the largest increase from 1993 to 2004 was identified in the aggressive behavior syndrome score (Cohen's *d *=* *.50), followed by the externalizing problem score (Cohen's *d *=* *.40) and CBCL total problem score (Cohen's *d *=* *.36), respectively (Table S4). Interestingly, a small but significant decrease was identified in the attention problem syndrome score between the 1993 and 2004 cohorts (Cohen's *d *=* *.34).

### Changes in clinical-level behavioral/emotional problems in children between 1993 and 2004

To examine whether the above findings held at a more clinical level, additional analyses were conducted with CBCL clinical (dichotomized) scores (Tables S5 and S6). From the 1993 to the 2004 cohort, the proportion of children in the clinical range for aggressive behavior increased almost fivefold, and the proportion of children in the clinical range for CBCL total problems and externalizing problems almost doubled. Other findings in support of the continuous outcome analyses included an increase in thought problem syndrome score and anxious/depressive problem syndrome scores. Fully adjusting for changes in maternal and child characteristics between the cohorts did not account for the increase in the observed rise of any of these scores.

## Discussion

Our study investigated an 11-year time trend in parent-reported behavioral and emotional problems among 4-year-old children in Brazil. General differences between the 1993 and 2004 Pelotas birth cohorts were identified in years of maternal schooling, marital status, prevalence of maternal psychiatric problems, primiparity, maternal employment, and proportion of preterm births. After fully accounting for these differences, significant increases in CBCL total problems, externalizing, aggressive, anxious/depressed, and thought problems remained evident in both the continuous and the dichotomized analyses. It must be noted, however, that the applicability of thought problems to 4-year olds is rarely clinically endorsed, and the significant change in this syndrome must thus be treated cautiously. From 1993 to 2004, aggressive behavior syndrome scores showed the largest increase, with a moderate effect size, followed by externalizing scores and CBCL total problem scores, respectively. These increases were largest in children from families with fewer assets and with mothers with less education.

This study is the first to document trends in children's mental health in a middle-income population and provides the first evidence for an increase in child emotional and behavioral problems, particularly a rise in aggressive behavior. It also has a number of methodological strengths. The comparisons rely on uniform modes of data collection (prospective information obtained among population-based samples in the same location with the same ethnic background), combined with the use of a standardized and well-validated instrument for children behavioral/emotional problems at both time points. Data collection was performed by highly trained fieldworkers supervised by the same psychologist, with high follow-up rates and low frequencies of missing data.

There are, however, a number of limitations to this study that must be considered. First, the 4-year assessment of behavioral and emotional problems in the 1993 cohort was based on a subsample rather than the whole cohort. To address this issue, the oversampling of low birthweight children was statistically accounted for in all analyses, as has been done in many previous studies on this cohort (Barros et al., 2012; Brion et al., 2010, 2011; Matijasevich et al., 2011; Victora et al., 1998), after which the subsample was shown not to differ from the whole cohort on any maternal or child characteristics. Furthermore, if anything, the oversampling of low birthweight children in the 1993 cohort would be expected to weaken the time trends identified by the current paper, as the prevalence of psychological problems in the 1993 cohort would be inflated by a large proportion of low birthweight children. It is, however, still important to consider possible differences arising from sampling differences when interpreting the current results. A second limitation is the possibility of reporting bias introduced by the use of maternal report in this study. Unfortunately, other sources of report, such an alternate caregiver or preschool teacher, were not available to compare with maternal reports in the 1993 and 2004 Pelotas birth cohorts. It is reassuring that there is evidence (Ivanova et al., 2010) that parents across many different societies were able to accurately rate their children's behavior, and that their descriptions of child behaviors converged to support six or seven basic behavior syndromes, supporting the use of problem checklists with parents from diverse backgrounds. It is possible, however, that as awareness and social acceptability of child behavioral problems have increased in the past decades, the rise in prevalence from 1993 to 2004 may partially reflect parental ability to recognize and willingness to report problematic behavior in their children. The fact that only some CBCL scales showed an increase over time, some remained unchanged, and some even decreased, suggests that our results are not the consequence of a broad societal increase in reporting sensitivity. A third limitation of this study was that maternal psychiatric problems were evaluated at different time points (4 years in the 1993 cohort and 3 months in the 2004 cohort). As maternal psychiatric problems were assessed at different time points, it is possible that the effect of this variable on child emotional and behavioral problems differed between cohorts. As expected, this pattern was identified in this study, with significant interactions in 7/11 outcomes (although, interestingly, the effect of maternal psychiatric problems on total CBCL scores did not differ). It is important to note, however, that across both cohorts, maternal psychiatric problems had the strongest effect of all the maternal and child characteristics on child emotional and behavioral problems. This suggests that in the current analysis, maternal psychiatric problems were an important confounder in the analysis of time trends in child emotional and behavioral problems, despite heterogeneity of time of assessment.

Our study identified a rise in behavioral and emotional problems among preschool children, particularly in aggressive behavior. These results suggest that, similar to high-income countries that have identified a recent increase in child behavioral problems (Achenbach et al., 2003), there has been an increase in these problems in a middle-income country. It should be noted, however, that not all studies in high-income countries have demonstrated a rise in childhood behavioral problems, with studies such as Tick et al. in Dutch 2- and 3-year olds (Tick et al., 2007b) and Maughan, Collishaw, Meltzer, & Goodman (2008) in British 5- to 15-year olds identifying small decreases in parent-reported psychological problems.

In generalizing the results of this study to Brazil as a whole, it must be noted that the city of Pelotas has several economic similarities and differences. The 11-year period between 1993 and 2004 saw political and economic changes in Brazil. The country has experienced economic growth from 2000 and several programs targeting the poorest population groups have been implemented. In spite of the economic improvement observed in the country, the Pelotas region had slower growth compared with the national average. In 2010, gross domestic product (GDP) per capita in Pelotas was US5976, lower than that observed for Brazil (*US* 8161). Nevertheless, substantial improvements in maternal health and education were observed in Pelotas during the study period (Barros et al., 2005), and the illiteracy rate in town for the year 2010 was considerably lower than for Brazil as a whole (4.1% vs. 8.7%, respectively). Furthermore, in 2011, the infant mortality rate for the city of Pelotas was 15.1 per 1000 live births, similar to that observed in Brazil (15.6 per 1000 live births). As such, even though our data are all from a single city, we feel that our results could be representative of children living in middle-size and urban cities in Brazil.

Several changes in family life, income, and family size have taken place across many different countries over the last few decades, and the differences in maternal and child characteristics between the 1993 and 2004 Pelotas birth cohorts indicate that Brazil is no exception. A study that examined how such changes affect adolescent mental health trends in the United Kingdom found that rising rates of adolescent antisocial behavior were only very modestly explained by concurrent changes in family type and socioeconomic conditions (Collishaw, Goodman, Pickles, & Maughan, 2007). In our study, the observed increase in child aggressive behavior was not accounted for by changes in familial, maternal, and child characteristics between 1993 and 2004. This suggests that something other than the sociodemographic covariates included in this study account for this increase.

It is plausible that changes in behavioral problems among children, especially among the poorest communities, could reflect broader changes in the local environment in terms of insecurity and violence. This study identified that the rise in child psychological problems was more marked in children from families with fewer assets and with less educated mothers. Neighborhood context variables, such as socioeconomic stratification, racial/ethnic segregation, and violence, have been shown to explain mental health disorders in adolescents (Aneshensel & Sucoff, 1996). Criminal indicators in Pelotas showed that the number of criminal offenses at least doubled between 2002 and 2011 (i.e., vehicle thefts and crimes related to the possession and traffic of arms and drugs) (Secretaria da Saúde Pública, 2002-2011), although data over the exact time period of our study were not available.

A second variable that may contribute to the explanation of the increase in aggressive child behaviors is paternal psychological problems, such as antisocial behavior. Paternal psychopathology has been shown to be associated with child behavioral problems, and is suggested to specifically affect externalizing problems (Phares & Compas, 1992). Despite including detailed data on a wide range of relevant sociodemographic covariates, paternal psychological state was not measured in this study.

This study has a number of important implications. Child psychological problems can have a long-lasting impact on later life and have been associated with later psychiatric problems, poor educational achievement, teenage parenthood, marital problems, and contact with the criminal justice system (Fergusson & Horwood, 2001; Goodman, Joyce, & Smith, 2011; Richards et al., 2009). As such, these outcomes are associated with major costs to the individuals, their families, and society, and may have profound policy implications. The increase in externalizing problems and aggressive behavior observed in our study, if replicated by other studies, is a public health concern and may have considerable implications for public health services.

In conclusion, this study of secular trends in child psychological problems, the first in a middle-income country, provides evidence that, between 1993 and 2004, there was an increase in psychological problems in Brazilian preschool children, particularly in aggressive behavior. This increase was not attributable to changes in broader social and demographic factors during this time period. Given the potential long-term effects of childhood psychological problems on an individual's life course, and if further research confirms the increases identified in this study, early identification of such problems needs to be a public health priority.

## Supporting Information

Table S1 Maternal and child characteristics in the 1993 (*n *=* *633) and 2004 (*n *=* *3,750) Pelotas cohort studies. **Table S2** Marginal means (*SE*) of significant main effects of familial, maternal, and child characteristics on CBCL scores…. **Table S3** *F* statistics of multivariable ANOVA cohort interaction effects (variable*cohort) of…. **Table S4** Effect sizes (Cohen's d and effect size *r*) of change in CBCL scores from 1993 to 2004. **Table S5** Percentages of CBCL clinical scores in the 1993 and 2004 Pelotas cohort studies adjusted for age (months)…. **Table S6** ANOVA analyses of differences in CBCL clinical scores between the 1993 and 2004 cohorts. **Figure S1** (a) Education*Externalizing score interaction. (b) Education*Internalizing score interaction. (c) Psychiatric*Externalizing score interaction. (d) Psychiatric*Internalizing score interaction.
