# Supplementary material for: Increase in child behavior problems among urban Brazilian 4-year olds: 1993 and 2004 Pelotas birth cohorts
Source: J Child Psychol Psychiatry. 2014 Apr 16;55(10):1125–34. doi: 10.1111/jcpp.12236 (PMC4263231; doi:10.1111/jcpp.12236)
Supplement: Table S1 — Maternal and child characteristics in the 1993 (n = 633) and 2004 (n = 3,750) Pelotas cohort studies. Table S2 Marginal means (SE) of significant main effects of familial, maternal, and child characteristics on CBCL scores…. Table S3 F statistics of multivariable ANOVA cohort interaction effects (variable*cohort) of…. Table S4 Effect sizes (Cohen's d and effect size r) of change in CBCL scores from 1993 to 2004. Table S5 Percentages of CBCL clinical scores in the 1993 and 2004 Pelotas cohort studies adjusted for age (months)…. Table S6 ANOVA analyses of differences in CBCL clinical scores between the 1993 and 2004 cohorts. Figure S1 (a) Education*Externalizing score interaction. (b) Education*Internalizing score interaction. (c) Psychiatric*Externalizing score interaction. (d) Psychiatric*Internalizing score interaction. [file jcpp0055-1125-SD1.docx]

**Online Supplemental Material**

**Increase in child behavior problems among urban Brazilian four-year-olds: 1993 and 2004 Pelotas Birth cohorts**

Table S1. Maternal and child characteristics in 1993 (n=633) and 2004 (n=3750) Pelotas cohort studies

| Variables | Pelotas 1993 | Pelotas 2004 | *x^2^* | *p-*value |
| --- | --- | --- | --- | --- |
| Assets index (quintiles)  1^st^  2^nd^  3^rd^  4^th^  5^th^ | 17.8  18.5  23.5  18.8  21.5 | 20.3  19.8  20.0  20.1  19.8 | 1.27 | 0.279 |
| Maternal schooling (years)  0-4  5-8  ≥9 | 27.3  45.2  27.5 | 15.1  41.8  43.2 | 35.26 | <0.001 |
| Marital status  With partner  Single mother | 89.5  10.5 | 84.2  15.8 | 10.64 | 0.001 |
| Maternal age (years)  ≤19  20-34  ≥35 | 13.4  74.0  12.6 | 18.8  67.5  13.7 | 5.37 | 0.005 |
| Maternal skin color  White  Black/mixed | 77.1  22.9 | 73.1  26.9 | 3.92 | 0.050 |
| Parity  0  1  ≥2 | 33.0  27.1  39.9 | 39.4  26.6  34.0 | 4.70 | 0.009 |
| Maternal smoking during pregnancy  No  Yes | 69.2  30.8 | 72.8  27.2 | 3.11 | 0.078 |
| Maternal employment  Never  Sometime  Always | 42.0  53.7  4.3 | 29.1  61.1  9.8 | 21.48 | <0.001 |
| Maternal psychiatric problems  No  Yes | 73.8  26.2 | 79.8  20.2 | 10.46 | 0.001 |
| Preterm birth (<37 weeks)  No  Yes | 90.4  9.6 | 86.3  13.8 | 10.28 | 0.001 |
| Low birthweight (<2500 g)  No  Yes | 90.2  9.8 | 90.9  9.1 | 0.72 | 0.397 |
| Child’s sex  Male  Female | 49.5  50.5 | 51.8  48.2 | 1.00 | 0.317 |
| Multiple pregnancy  No  Yes | 98.6  1.4 | 98.0  2.0 | 1.11 | 0.291 |

*Note*: *χ^2^* test for the difference between 1993 (weighted) and 2004 Pelotas cohort studies.

Table S2. Marginal means (SE) of significant main effects of familial, maternal and child characteristics on CBCL scores, across both cohorts, adjusted for age at time of testing

| Variables | CBCL Total | Internalising | Externalising | Withdrawn | Somatic | Anx/Dep | Social | Thought | Attention | Rule.break | Aggressive |
| --- | --- | --- | --- | --- | --- | --- | --- | --- | --- | --- | --- |
|  |  |  |  |  |  |  |  |  |  |  |  |
| Maternal schooling (years)  0.4  5.8  ≥9 | .  .  . | 7.5 (0.2)  6.6 (0.1)*  5.3 (0.1)* | .  .  . | .  .  . | .  .  . | 3.9 (0.1)  3.3 (0.1)*  2.6 (0.1)* | 2.3 (0.1)  1.9 (0.1)*  1.5 (0.1)* | .  .  . | .  .  . | .  .  . | .  .  . |
| Marital status  With partner  Single mother | 33.0 (0.3)  37.0 (0.6)* | 6.1 (0.1)  7.0 (0.2)* | 14.7 (0.1)  16.8 (0.3)* | 2.3 (0.1)  2.6 (0.1)* | .  . | 3.1 (0.1)  3.5 (0.1)* | .  . | .  . | .  . | .  . | 12.3 (0.1)  13.8 (0.2)* |
| Maternal age (years)  ≤19  20.34  ≥35 | 37.4 (0.6)  33.4 (0.3)*  31.1(0.7)* | .  .  . | 16.6 (0.3)  14.9 (0.1)*  13.7 (0.3)* | 2.6 (0.1)  2.3 (0.1)*  2.1 (0.1)* | .  .  . | .  .  . | 2.0 (0.1)  1.8 (0.1)*  1.6 (0.1)* | .  .  . | .  .  . | .  .  . | 13.7 (0.2)  12.4 (0.1)*  11.5 (0.2)* |
| Maternal skin color  White  Black/mixed | .  . | .  . | .  . | .  . | .  . | .  . | .  . | .  . | 2.6 (0.1)  3.1 (0.1)* | .  . | .  . |
| Parity  0  1  ≥2 | .  .  . | 6.2 (0.1)  5.8 (0.1)  6.6 (0.1)* | .  .  . | .  .  . | .  .  . | 3.2 (0.1)  2.9 (0.1)*  3.3 (0.1)* | .  .  . | .  .  . | 2.8 (0.1)  2.6 (0.1)  2.8 (0.1)* | .  .  . | .  .  . |
| Maternal smoking during pregnancy  No  Yes | Maternal smoking  31.8 (0.3)  38.8 (0.5)* | Maternal smoking  .  . | Maternal smoking  14.1 (0.1)  17.4 (0.2)* | .  . | .  . | .  . | 1.7 (0.1)  2.1 (0.1)* | 0.4 (0.1)  0.6 (0.1) | 2.5 (0.1)  3.4 (0.1)* | 2.3 (0.1)  3.1 (0.1)* | 11.8 (0.1)  14.2 (0.2)* |
| Maternal employment  Never  Sometimes  Always | .  .  . | .  .  . | .  .  . | .  .  . | .  .  . | .  .  . | 2.0 (0.1)  1.7 (0.1)*  1.6 (0.1) | 0.5 (0.1)  0.5 (0.1)  0.4 (0.1) | .  .  . | .  .  . | .  .  . |
| Maternal psychiatric problems  No  Yes | 31.0 (0.3)  43.4 (0.5)* | .  . | .  . | .  . | 0.7 (0.1)  1.2 (0.1)* | .  . | 1.6 (0.1)  2.5 (0.1)* | .  . | .  . | 2.3 (0.1)  3.3 (0.1)* | .  . |
| Low birthweight (<2500 g)  No  Yes | .  . | .  . | .  . | .  . | .  . | .  . | .  . | .  . | .  . | .  . | .  . |
| Child’s sex  Male  Female | .  . | .  . | 15.3 (0.2)  14.8 (0.2)* | .  . | .  . | 3.0 (0.1)  3.2 (0.1)* | .  . | .  . | 2.9 (0.1)  2.5 (0.1)* | 2.7 (0.1)  2.3 (0.1)* | .  . |
| Multiple pregnancy  No  Yes | .  . | .  . | .  . | .  . | .  . | .  . | 1.79 (0.1)  1.78 (0.1) | 0.5 (0.1)  0.4 (0.1) | .  . | .  . | .  . |
| *Note.* *x^2^* test for the difference between 1993 (weighted) and 2004 Pelotas cohort studies. Marginal means presented for main effects that were not involved in a significant interaction; *indicates significantly different to category above, adjusted for multiple comparisons using Bonferroni adjustment. | | | | | | | | | | | |

| Table S3. *F* statistics of multivariable ANOVA cohort interaction effects (variable*cohort) of familial, maternal and child characteristics on CBCL scores | | | | | | | | | | | |  |
| --- | --- | --- | --- | --- | --- | --- | --- | --- | --- | --- | --- | --- |
|  | CBCL Total | Internalising | Externalising | Withdrawn | Somatic | Anx/Dep | Social | Thought | Attention | Rule-breaking | Aggressive | |
|  |  |  |  |  |  |  |  |  |  |  |  | |
| Family assets | 3.88* | 1.47 | 5.10** | 1.67 | 0.09 | 1.85 | 1.84 | 0.31 | 3.23* | 5.90** | 3.83* | |
| Maternal schooling | 3.24* | 0.39 | 3.92* | 0.49 | 0.34 | 0.17 | 2.06 | 3.70* | 3.25* | 6.26* | 3.40* | |
| Marital status | 0.20 | 1.90 | 1.45 | 2.57 | 0.25 | 3.07 | 0.08 | 0.04 | 0.09 | 0.01 | 2.55 | |
| Maternal age | 1.08 | 1.14 | 0.53 | 1.23 | 0.24 | 0.86 | 2.12 | 0.29 | 1.22 | 1.71 | 0.91 | |
| Maternal skin colour | 0.27 | 0.01 | 0.56 | 0.01 | 0.10 | 0.13 | 0.25 | 0.12 | 0.14 | 0.91 | 0.13 | |
| Parity | 0.17 | 1.26 | 1.21 | 4.52* | 0.18 | 0.12 | 1.17 | 0.47 | 1.16 | 0.17 | 0.78 | |
| Maternal smoking | 0.59 | 2.10 | 2.83 | 0.08 | 0.42 | 8.23* | 0.50 | 0.33 | 3.38 | 0.52 | 2.82 | |
| Maternal employment | 0.59 | 0.06 | 1.01 | 0.26 | 2.05 | 0.39 | 0.47 | 1.61 | 0.04 | 1.58 | 0.99 | |
| Maternal psychiatric | 3.66 | 6.52* | 8.27** | 11.61* | 3.41 | 6.94* | 0.05 | 4.94* | 4.25* | 1.51 | 6.83* | |
| Preterm birth | 0.44 | 0.21 | 0.14 | 0.01 | 1.52 | 0.11 | 0.14 | 0.03 | 0.08 | 0.03 | 0.04 | |
| Low birthweight | 0.79 | 0.01 | 1.12 | 0.54 | 0.23 | 0.05 | 0.24 | 0.68 | 4.46* | 0.26 | 1.15 | |
| Child’s sex | 0.01 | 0.23 | 0.59 | 0.46 | 0.12 | 1.00 | 0.01 | 0.01 | 2.51 | 0.94 | 0.13 | |
| Multiple pregnancy | 0.70 | 1.99 | 1.00 | 0.01 | 7.62* | 0.99 | 2.88 | 0.51 | 0.87 | 0.03 | 1.25 | |
| Note. Controlling for age at follow-up and using weighted 1993 scores; *p<.05 **p<.001 | | | | | | | | | | | |  |


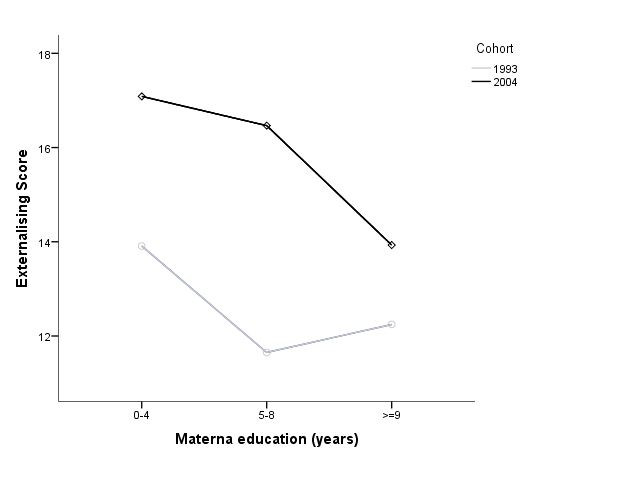


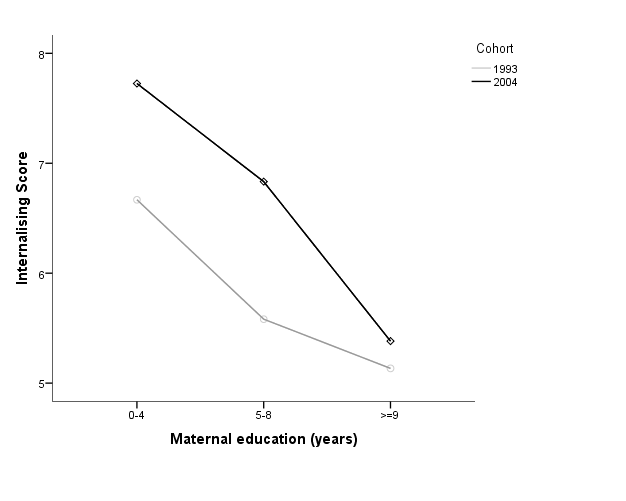


Figure S1a. Education*Externalising score interaction Figure S1b. Education*Internalising score interaction


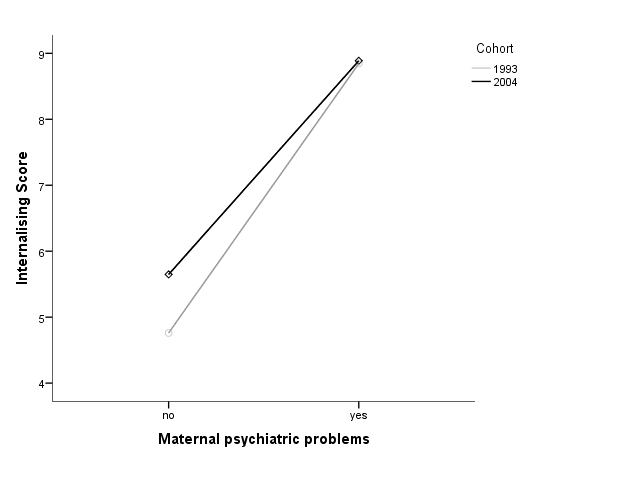

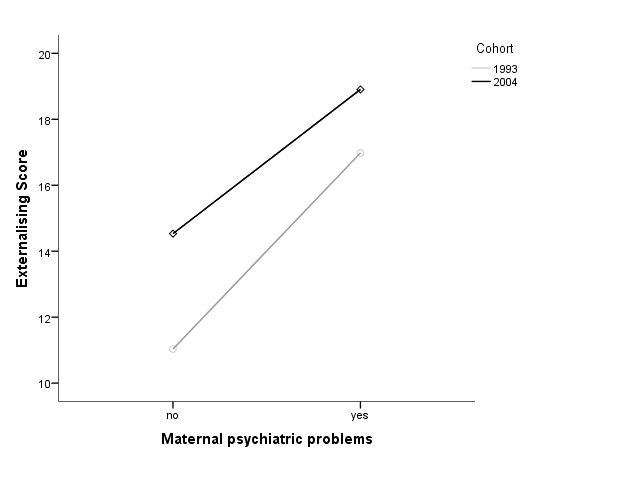


Figure S1c. Psychiatric*Externalising score interaction Figure S1d. Psychiatric*Internalising score interaction

Table S4. Effect sizes (Cohen’s d and effect size *r*) of change in CBCL scores from 1993 to 2004

| CBCL Scales | Cohen’s *d* | Effect size *r* |
| --- | --- | --- |
|  |  |  |
| Total Problems | 0.363** | 0.089** |
| Internalising | 0.033* | 0.008* |
| Externalising | 0.399** | 0.098** |
| Withdrawn | 0.060 | 0.015 |
| Somatic | 0.171** | 0.042** |
| Anxious/Depressed | 0.009 | 0.002 |
| Social | 0.040 | 0.010 |
| Thought | 0.102** | 0.025** |
| Attention | 0.336** | 0.083** |
| Rule-breaking | 0.058 | 0.014 |
| Aggressive | 0.503** | 0.123** |
|  |  |  |

Note. *p<.05; **p<.001. Corrected for uneven group size.

| Table S5 Percentages of CBCL clinical scores in 1993 and 2004 Pelotas cohort studies adjusted for age months at the time of measurement | | | | | | |
| --- | --- | --- | --- | --- | --- | --- |
| CBCL scales | Pelotas 1993  (n= 633) | | Pelotas 2004  (n=3750) | | χ^2^ | *p* |
|  | % | 95% CI | % | 95% CI |  |  |
|  |  |  |  |  |  |  |
| Total problems | 14.3 | 0.9-17.7 | 22.2 | 20.8-23.6 | 10.86 | .001* |
| Internalizing | 5.6 | 3.4-7.7 | 7.8 | 6.9-8.7 | 0.15 | .695 |
| Externalizing | 18.2 | 14.4-21.9 | 30.1 | 28.5-31.6 | 21.44 | <.001** |
| Withdrawn | 2.6 | 0.7-4.6 | 1.5 | 1.1-1.9 | 5.98 | .014* |
| Somatic complains | 0.4 | 0.0-0.8 | 0.7 | 0.4-0.9 | 0.31 | .575 |
| Anxious/ Depressed | 0.4 | 0.0-0.7 | 1.0 | 0.7-1.3 | 0.17 | .677 |
| Social problems | 0.5 | 0.0-1.1 | 1.0 | 0.6-1.3 | 0.12 | .728 |
| Thought problems | 0.3 | 0.0-0.6 | 1.4 | 1.0-1.7 | 1.08 | .298 |
| Attention problems | 1.2 | 0.4-2.0 | 0.9 | 0.6-1.2 | 5.12 | .019* |
| Rule-Breaking Behaviour | 6.8 | 4.2-9.5 | 6.1 | 5.3-6.9 | 0.01 | .988 |
| Aggressive Behaviour | 2.1 | 0.8-3.4 | 9.3 | 8.3-10.3 | 27.54 | <.001** |

*Note*: CI, confidence intervals; *p<.05; **p<.001

| Table S6. ANOVA analyses of differences in CBCL clinical scores between 1993 and 2004 cohorts | | | | | | | | | | | | | | |
| --- | --- | --- | --- | --- | --- | --- | --- | --- | --- | --- | --- | --- | --- | --- |
| CBCL Scales | Model 1 | |  | Model 2 | |  | Model 3 | |  | Model 4 | |  | Model 5 | |
|  | F | *p* |  | F | *p* |  | F | *p* |  | F | *p* |  | F | *p* |
|  |  |  |  |  |  |  |  |  |  |  |  |  |  |  |
| Total Problems | 13.61 | <.001** |  | 16.73 | <.001** |  | 14.82 | <.001** |  | 14.50 | <.001** |  | 16.40 | <.001** |
| Internalising | 2.80 | .094 |  | 3.91 | .048* |  | 3.46 | .063 |  | 3.55 | .060 |  | 3.02 | .082 |
| Externalising | 25.33 | <.001** |  | 29.12 | <.001** |  | 26.42 | <.001** |  | 26.18 | <.001** |  | 28.95 | <.001** |
| Withdrawn | 2.69 | .101 |  | 1.48 | .224 |  | 1.85 | .174 |  | 1.75 | .187 |  | 4.50 | .034* |
| Somatic | 0.55 | .460 |  | 0.33 | .568 |  | 0.29 | .590 |  | 0.29 | .592 |  | 0.44 | .508 |
| Anxious/Depressed | 3.28 | .070 |  | 4.39 | .036* |  | 4.58 | .032* |  | 4.73 | .030* |  | 5.99 | .014* |
| Social | 1.33 | .248 |  | 1.75 | .186 |  | 1.62 | .203 |  | 1.74 | .187 |  | 0.91 | .341 |
| Thought | 8.41 | .004* |  | 8.19 | .004* |  | 7.79 | .005* |  | 8.06 | .005* |  | 5.55 | .019* |
| Attention | 0.61 | .435 |  | 0.34 | .562 |  | 0.35 | .553 |  | 0.27 | .601 |  | 0.09 | .763 |
| Rule-breaking | 0.32 | .574 |  | 0.02 | .904 |  | 0.09 | .760 |  | 0.10 | .754 |  | 0.04 | .850 |
| Aggressive | 29.27 | <.001** |  | 30.31 | <.001** |  | 28.46 | <.001** |  | 28.59 | <.001** |  | 27.90 | <.001** |
|  |  |  |  |  |  |  |  |  |  |  |  |  |  |  |
| Note. *p<.05; **p<.001  Model 1. Adjusted for age at time of CBCL assessment  Model 2. Adjusted for model 1 plus family socioeconomic status (assets index and maternal education)  Model 3. Adjusted for model 2 plus maternal characteristics (marital status, maternal age, parity, maternal smoking during pregnancy)  Model 4. Adjusted for model 3 plus child characteristics (low birthweight and multiple pregnancy)  Model 5. Adjusted for model 4 plus maternal employment and maternal psychiatric problems | | | | | | | | | | | | | | |
